# The Borderland of Consumption

**Published:** 1905-01

**Authors:** 


### THE BORDERLAND OF CONSUMPTION.*

The author took up the subject of early diagnosis of tuberculosis, its curability and its prevention, urging that the medical profession needs better training in the field of early symptomology of consumption; and the public in general education on the subject of the hygiene of the disease.

Tuberculosis is still the most widely scattered, the most prevalent, and the *one* perpetual plague universally. It still consigns to the tomb more people than the slaughter of war in all its atrocious forms. The death-roll of all the wars of the nineteenth century is estimated at 14,000,000 (1) and that of consumption in the same period and countries at 30,000,000.

Our country's yearly death-rate by it is approximated at 160,000.

---

*Abstract of Paper read by Paul Paquin, M.D., Asheville, N. C., before the Ohio Valley Medical Society, November, 1904.

Of these invalids perhaps one-fifth are not wage earners and have incomes continuing while sick. The remaining four-fifths lose their previous daily revenue. If we estimate that all of the latter are too ill to earn anything for one year, and that each of them was previously earning one dollar a day three hundred days a year, what does it represent in yearly loss? $38,400,000. If the average length of invalidism is two years it means a loss of $76,800,000 a year. These are flat losses of income.

Why is the world so strangely apathetic with regard to this, the very gravest and most comprehensively destructive of all the ills of mankind? First, because the majority of the medical profession continues to consider tuberculosis, particularly the pulmonary form, as incurable in any event. Second, because that circumstances and the lack of instruction in sanitary science in our system of education has produced a public mental attitude of wanton optimism. Third, because there remain here and there a few who reject facts of infection mathematically demonstrated the world over and continue a public crusade in favor of their mistaken ideas. Fourth, because when tuberculosis is recognized the truth is often concealed so long from the patient and relatives that the former loses precious chances of recovery and is allowed to disseminate the disease broadcast. Fifth, because, by the marriage of consumptives, prolific new centers of infection with far-reaching fatal influences reinforce the popular idea of unavoidable pathogenic perpetuity. Sixth, because the diagnosis of the disease, in the great majority of cases, is made too late for any measure of prevention or cure to be effective.

Yet tuberculosis is proven largely curable by the experience of specialists, general practitioners and experimentalists. How are we to remedy the situation, cure more cases and finally stamp out the disease? On this question he suggests a more complete medical education so that early diagnosis may be possible.

The medical college that will establish a competent chair to teach the pathology and clinical factors of the borderland of disease will confer a blessing on the profession and humanity, for it will do immediate good and soon be emulated. This sphere of medical science, including physiologic conditions as they taper down and change to pathologic states, and which have been explored only by few, contains the very germ of knowledge for the early diagnosis of all diseases affecting the constitution and could be treated in a most interesting manner without in any way interfering, but rather supporting or supplementing the regular course on the principles and practice of medicine. A dozen or two of good practical lectures would be of immense advantage to students and physicians alike.

The author argues that tuberculosis is, after all, a disease of nutrition due to a faulty nervous system which permits directly and indirectly the introduction and development of germs and their toxins in the system and claims that, under a complete and proper control and guidance of all the organs in the system, consumption could not occur.

Following other writers and practitioners the author believes that the tonsils and other tissues of the neighborhood constitute the chief gateway of the tubercle bacilli and mentions tonsillitis, habitual nose-bleed, chronic hacking cough, pleurisies, deficient nutritions and dyspepsias, etc., as early symptoms of consumption in the borderland.

Great stress was laid on the necessity of diagnosing the borderland of tuberculosis in order to arrest the malady in its incipiency, on the usefulness of open-air life in a suitable climate, the value of certain remedies in the line of natural law, as certain vaccines and serums when applicable and in select suitable cases, and deprecates the pitiful habit of sending consumptives away moribund, often without any prior arrangement with a physician where they are sent to care for them, save them from errors of conduct and treatment in a new climate and the pitfalls incident to new, strange surroundings.—*The Louisville Monthly Journal of Medicine and Surgery.*

